# Gcn5 histone acetyltransferase is present in the mitoplasts

**DOI:** 10.1242/bio.041244

**Published:** 2019-02-15

**Authors:** Arianna Montanari, Manuela Leo, Veronica De Luca, Patrizia Filetici, Silvia Francisci

**Affiliations:** 1 Department of Biology and Biotechnologies “Charles Darwin”, Sapienza University of Rome, Piazzale Aldo Moro 5, 00185 Rome, Italy; 2 Pasteur Institute Italy - Cenci Bolognetti Foundation, Sapienza University of Rome, Viale Regina Elena 291, 00161 Rome, Italy; 3 Institute of Molecular Biology and Pathology - CNR, Sapienza University of Rome, Piazzale Aldo Moro 5, 00185 Rome, Italy

**Keywords:** Yeast, Respiration, Mitochondria, Mitochondrial DNA, Lysine-acetyltransferase Gcn5

## Abstract

In *Saccharomyces cerevisiae* the Lysine-acetyltransferase Gcn5 (KAT2) is part of the SAGA complex and is responsible for histone acetylation widely or at specific lysines. In this paper we report that *GCN5* deletion differently affects the growth of two strains. The defective mitochondrial phenotype is related to a marked decrease in mtDNA content, which also involves the deletion of specific regions of the molecule. We also show that in wild-type mitochondria the Gcn5 protein is present in the mitoplasts, suggesting a new mitochondrial function independent from the SAGA complex and possibly a new function for this protein connecting epigenetics and metabolism.

## Abstract (precis)

**Summary:** In yeast mitochondria the Gcn5 protein is present in the mitoplasts and is localized in the inner mitochondrial membrane. Its deletion affects the mitochondrial phenotype and is related to a marked decrease of mitochondrial DNA content.

## INTRODUCTION

Histone acetylation, the major chromatin modification regulating nuclear transcription, is a transient epigenetic mark, and is regulated by histone acetyltransferases and deacetylases. The K-acetyltransferase (KAT)-General Control Nonderepressible (Gcn5) has a major role in histone H3 acetylation at genome-wide level and at specific loci linked to stress response (Gaupel et al., 2015).

Several transcription factors and their cofactors target the histone acetylation complexes to distinct genes to facilitate either transcription or silencing.

In yeast, SAGA (Spt, Ada, Gcn5-acetyltransferase complex) is the main acetylation complex and is composed of two multiprotein modules having different catalytic activity: HAT for acetylation and DUB for deubiquitylation. Recently we have demonstrated that the ubiquitin protease 8 (Ubp8) of the DUB module is necessary in respiratory metabolism and we showed its expression is upregulated in respiratory condition compared to fermentation (Leo et al., 2018).

We have previously shown that the KAT2-Gcn5 protein of the HAT module is required for respiratory metabolism and oxygen consumption, indicating a role of this protein in mitochondria. Moreover, compared with fermentative growth, in respiratory condition the expression level of *GCN5* is upregulated at protein as well as at mRNA level (Canzonetta et al., 2016).

Studies on yeast mitochondrial proteome have previously shown the existence of a mitochondrial acetyltransferase Nat2 (Sickmann et al., 2003) but no further data have been reported on its mitochondrial role. Lysine acetylation of non-histone mitochondrial proteins has been reported in mammalian cells (Fernandes et al., 2015; Lu et al., 2015). In mouse, the acetyltransferase MOF has been shown to be present in nuclei and in mitochondria, and to control the expression of respiratory genes from both nuclear and mitochondrial (mt) DNA (Chatterjee et al., 2016).

For a long time mtDNA was considered to be naked – lacking histones – and therefore unprotected and vulnerable to damage. On the contrary, it has now been clarified that mtDNA is protein-coated and packaged into aggregates called nucleoids (Chen and Butow, 2005; Kukat and Larsson, 2013). However, H2A and H2B have been found to be present in the mammalian mitochondrial outer membrane (Choi et al., 2011). Histone-like proteins were found to bind mtDNA of several mammalian tissues (Kutsyi et al., 2005); these proteins might be acetylated but no report is available yet.

In this paper we report the presence of Gcn5 protein inside mitochondria as shown by western blot and supported by physiological, genetic and microscopic analysis. In particular we show that Gcn5 is involved in mtDNA maintenance: the deletion of GCN5 gene produces a marked decrease of mtDNA copy number and deletion of specific regions of the molecule. Moreover, we report important differences in the phenotype due to the *GCN5* deletion in two strains having different physiology and mtDNA organization.

These results allow a deeper understanding of the interactions between mitochondrial and nuclear genomes and suggest the involvement of mitochondrial activity in impacting the epigenetic landscape.

## RESULTS

We have previously demontrated that W303-1A cells deleted of the GCN5 gene show a thermosensitive phenotype (Vernarecci et al., 2008) and a reduced growth in glycerol containing medium at 28°C. Moreover, *GCN5* is required for efficient respiration and the gene is overexpressed in respiring cells compared with cells grown in fermentative condition; the level of the protein is two times higher in respiring cells (Canzonetta et al., 2016). To better understand the role of Gcn5 as a factor involved in respiratory metabolism, we now report the effect of *GCN5* deletion in two yeast strains with distinct origin and important differences in mtDNA structure and molecular weight.

### The studied *gcn5*Δ strains have different respiratory phenotypes

Laboratory yeast strains are genetically and physiologically heterogeneous, with differences in growth behaviours (Montanari et al., 2008; 2011; De Luca et al., 2006; 2009). W303-1A has been selected in French laboratories due to its good sporulation and formation of complete tetrads, which is useful for genetic analysis in which high respiration is necessary. D273-10B/A1 is an industrial strain mainly used in USA with good fermentative capability.

Fermentative and respiratory metabolism can be easily studied in *S. cerevisiae*; without functional mitochondria the cells are not able to grow on glycerol containing medium but can grow on a fermentable carbon source, such as glucose.

**Fig. 1.**
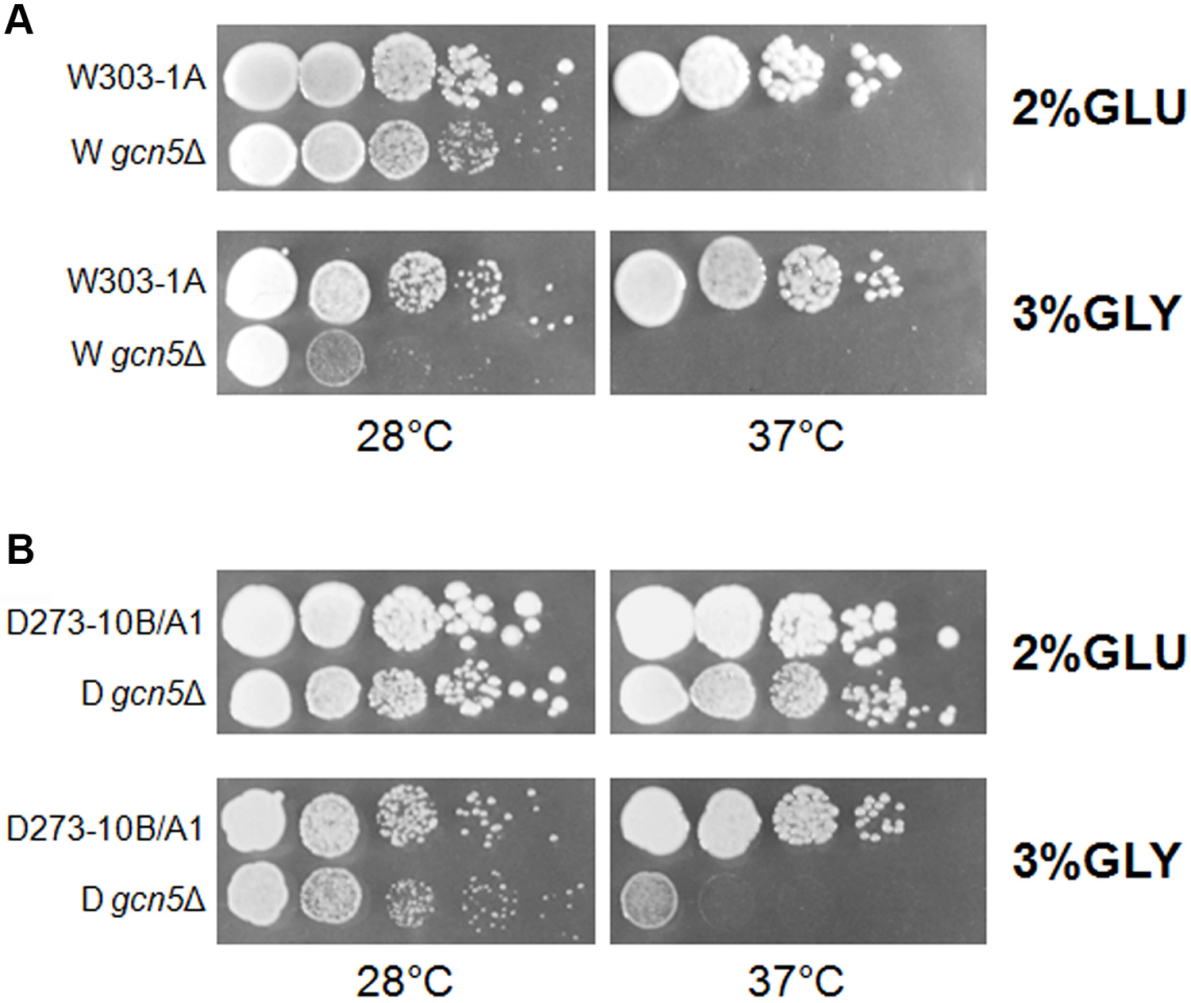
**Deletion of *GCN5* differently affects fermentative and respirative growth.** (A,B) Serial dilutions of two WT [W303-1A (A) and D273-10B/A1 (B)] and their derivate *gcn5*Δ strains grown overnight in YP 2% glucose containing medium were spotted on YP 2% glucose or 3% glycerol containing media and incubated at 28 or 37°C.

The effect of the deletion of the GCN5 gene on growth in fermentative or respiratory media in the above strains has been compared (Fig. 1A,B). Results indicate that W303-1A cells are more sensitive to *GCN5* deletion; the deletant acquires a thermosensitive phenotype in both metabolic conditions. On the contrary, in D273-10B/A1 cells, *GCN5* deletion does not result in defective growth in fermentative condition and growth in glycerol containing medium is impaired only at 37°C.

### A specific cross reveals an involvement between Gcn5 and mtDNA

**Fig. 2.**
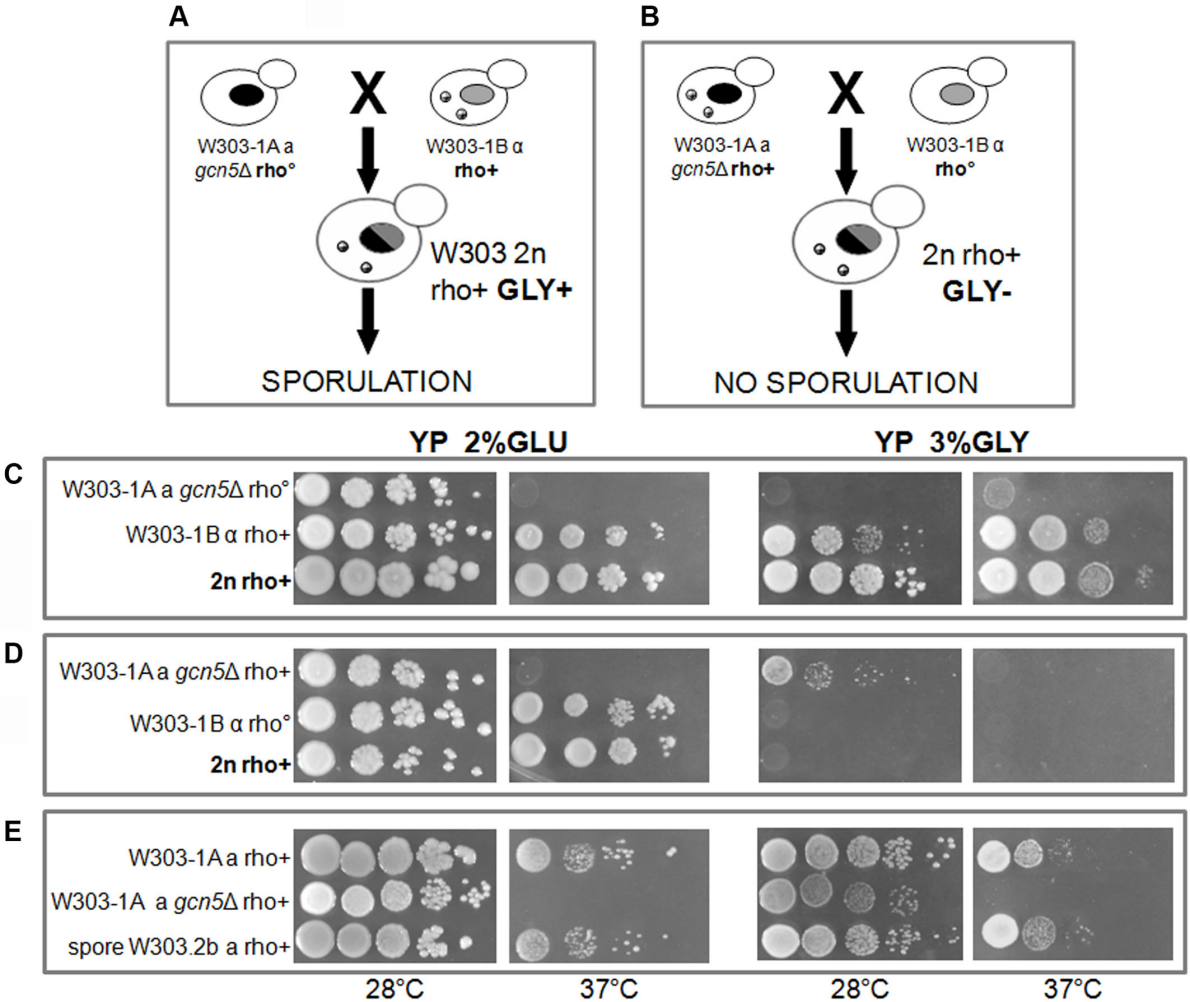
**Cross experiment demonstrates the relationship between mtDNA and Gcn5.** To obtain diploid competent for respiration it is required that the mtDNA does not derive from *gcn5*Δ strain. (A,B) Schematic procedure of the two crosses to reintroduce GCN5 gene in the deleted strain. Nucleus with wild-type chromosomal DNA is grey whereas in the presence of *GCN5* deletion it is black. MtDNA is shown as small beads; GLY+ or − indicate the capability to grow in glycerol containing medium. (C,D) Serial dilutions of parental strains and diploids of the crosses described in A and B, respectively. (E) Serial dilutions of WT, *gcn5*Δ and spore W303.2b (obtained from the cross described in A) in which the wild-type phenotype is completely restored. All strains are spotted on YP 2% glucose or in 3% glycerol containing media and incubated at 28 or 37°C.

In Fig. 2 we report the genetic experiment demonstrating the important correlation between mtDNA and Gcn5. The figure shows that the defective phenotype of the W303-1A *gcn5*Δ mutant is fully rescued by the reintroduction of *GCN5* by cross. As shown in Fig. 2, we obtained the diploids in two ways, crossing haploid cells *gcn5*Δ (nuclei indicated in black) with haploid cells retaining the *GCN5* gene (nuclei indicated in grey). Each cross was alternately performed between rho^+^ cells (with wild-type mtDNA) and rho° cells (without mtDNA) bearing the two different nuclear backgrounds (mtDNA is indicated with gray and white small beads). It is important to note that we only obtained respiring diploid cells able to grow on glycerol containing medium that were competent for sporulation in the cross between *gcn5*Δ rho° and W303-1B rho^+^ (Fig. 2A), but not in the cross between gcn5Δ rho^+^ and W303-1B rho° (Fig. 2B). The growth of the parental strains, the diploids and the spore is shown in Fig. 2C,E (first cross), D (second cross). This result shows that although the diploids from the two crosses have the same mitochondria and mtDNA, the sporulation was not obtained when mtDNA came from cells bearing *GCN5* deletion (Fig. 2B) and was not due to different levels of *GCN5* between rho^+^ and rho° cells (Fig. S1). Therefore, we might suggest a physical interaction between mtDNA and the Gcn5 protein.

### Effects of *GCN5* deletion on mtDNA

**Fig. 3.**
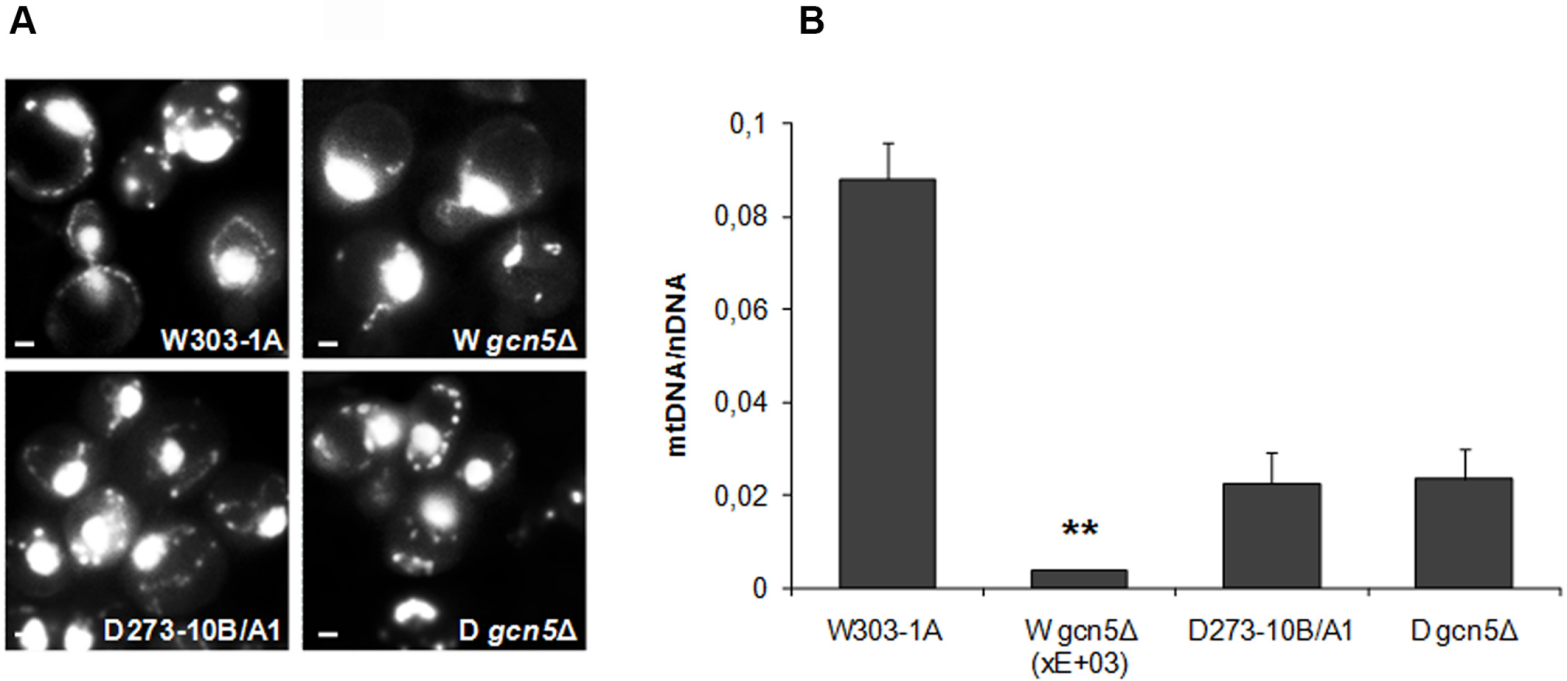
**Different effects of *GCN5* deletion in the strains W303-1A and D273-10B/A1*.*** (A) Fluorescence microscopy of DAPI staining of WT (W303-1A and D273-10B/A1) and of their derivate deleted mutants (W *gcn5*Δ and D *gcn5*Δ) grown overnight in YP 2% glucose containing medium. Scale bars: 0.5 μm. (B) qRT-PCR analysis of mtDNA level of the two WT and their *gcn5*Δ derivative strains as above, grown in YP 2% glucose containing medium. The ratio between nuclear DNA mean value and mtDNA mean value (*OXI1*/*ACT1*) was used to overcome the variability among samples caused by total DNA quality. Data derive from at least three independent experiments and statistical significance by Student's *t*-test is indicated. **P<0.01 for deleted versus WT strain.

To visualize the effects of the deletion in the two strains, we first performed DAPI staining to visualize the mtDNA. Results reported in Fig. 3A show that in the W303-1A *gcn5*Δ strain the number of fluorescent dots is strongly reduced compared to wild type (WT); on the contrary, no effect from deletion is observed in the D273-10B/A1 nuclear background.

We then examined, by RT-PCR, the level of mtDNA in WT and *gcn5*Δ strains. Results reported in Fig. 3B show that the ratio of mtDNA to nuclearDNA is strongly decreased (50 times) in the W303-1A mutant cells compared to WT cells; this defect was not observed in the D273-10B/1A cells. The different level of mtDNA in the two *gcn5*Δ strains is consistent with their different phenotypes and with the higher respiratory competence of W303-1A compared to D273-10B/A1 cells.

### Dynamics of mtDNA depletion during cell duplication indicates the loss of specific regions

We analysed the depletion of mtDNA in W *gcn5*Δ mutant strain to evaluate the relationship between the decrease of mtDNA copy number and cell duplication. We subcloned a large single fresh colony grown on YP 2% glucose plate four times, as reported in Table 2.

**Fig. 4.**
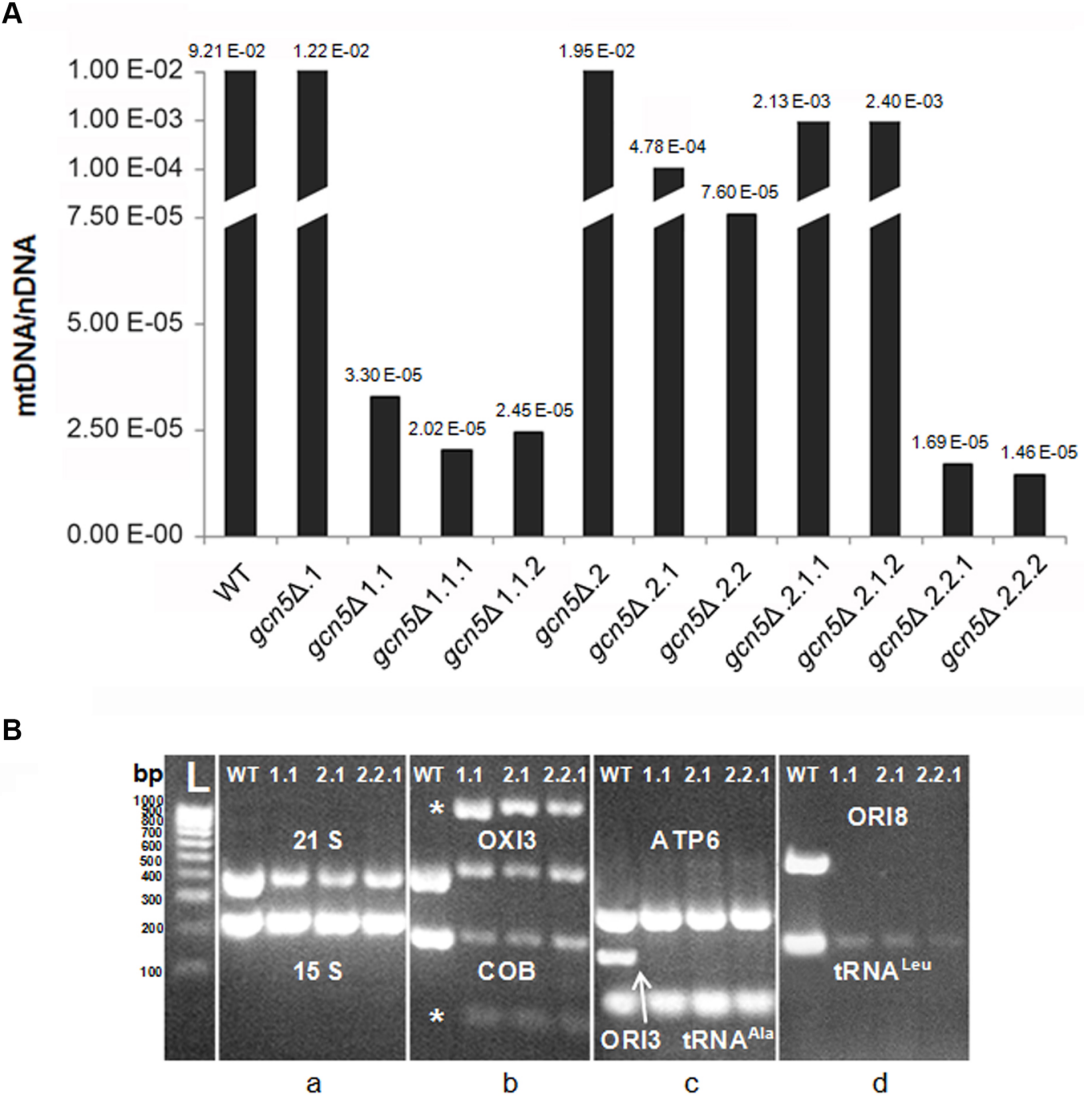
**In *gcn5*Δ strains, the decrease of mtDNA copy number is accompanied by deletion of specific regions of the molecule.** (A) qRT-PCR analysis of mtDNA copy number of the WT (W303-1A) and W *gcn5*Δ subclones reported in Table 2, grown in YP 2% glucose containing medium. The ratio between nuclear DNA mean value and mtDNA mean value (*OXI1*/*ACT1*) was used to overcome the variability among samples caused by total DNA quality. (B) Electrophoretic analysis on 1X TBE, 2.5% agarose gel of amplified sequences of mtDNA from WT (W303-1A) and from three W *gcn5*Δ clones is reported in Table 2. Amplified genes indicated in white. *, unspecific amplified sequence from COB and OXI3 genes.

Analysis of mtDNA copy number by qRT-PCR reveals that at the first generation the mtDNA content in the deleted mutant is already very low and decreases in the subclones, although the cells do not become rho° (Fig. 4A). In order to verify if the depletion of mtDNA could be related to the deletion of specific regions of the molecule, we performed different amplifications of mtDNA (70–500 bp) from WT and from all the W *gcn5*Δ clones reported in Table 2. The amplified regions were chosen to uniformly cover the entire mtDNA molecule. Fig. 4B is representative of the mtDNA PCRs of three clones. The absence of *GCN5* produces specific deletions in the mtDNA molecule: the amplifications of the origin of replication (ORI3 and ORI8) are absent (Fig. 4Bc,d), while the amplification levels of the 21S, COB, OXI3 and tRNA^Leu^ genes are decreased compared to the WT (Fig. 4Ba,b,d). Moreover, the presence of non-specific amplifications in the mtDNA of *gcn5*Δ clones suggests the presence of rearrangement regions including COB and OXI3 genes (Fig. 4Bb). The regions containing the ATP6, 15S and tRNA^Ala^ genes are always maintained in the mutant clones as in the WT (Fig. 4Ba,c, respectively).

### Colocalization of Gcn5 with a mitochondrial protein

**Fig. 5.**
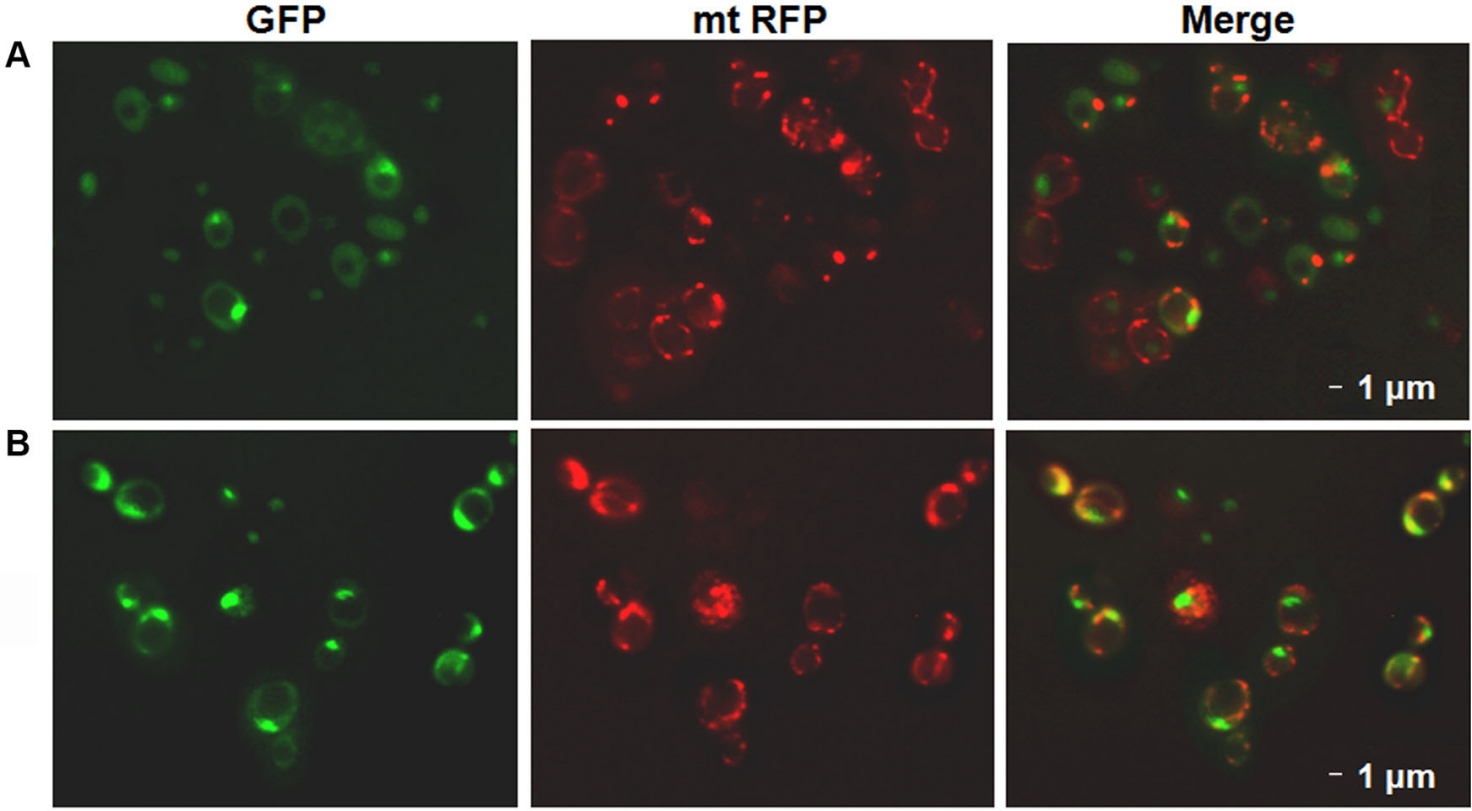
**GFP fluorescence colocalization** **of Gcn5.** (A,B) Fluorescence microscopy of the GCN5-GFP with the endogenous *GCN5* fused with the GFP gene grown in YP 2% glucose (A) or 3% glycerol (B) containing media to stationary phase. The fluorescent signals of the GCN5-GFP clone transformed with pmtRFP plasmid overexpressing the mitochondrial Red Fluorescent Protein show colocalization between Gcn5 and mitochondria.

To visualize the presence of Gcn5 in the mitochondria, we used a stationary culture of the commercial strain harbouring the GFP gene fused with the 3′-end of the GCN5 gene (GCN5-GFP clone, Life Technologies) (Huh et al., 2003), which was transformed with plasmid containing the Red Fluorescent Protein fused with the mitochondrial pre-sequence of subunit 9 of *Neurospora crassa* ATP-ase (pmtRFP) (Westermann and Neupert, 2000). Fig. 5 shows that the localization of the GFP signal of the fusion product is certainly nuclear, but it is also possible to see a speckled, less uniform localization in the cytoplasm, compatible to a mitochondrial localization. The merging signal obtained by the mtRFP is coherent with a partial colocalization of the GCN5-GFP fluorescence with mitochondria. The same results are obtained using cells grown in glucose or in glycerol containing media (Fig. 5A,B, respectively). The results of fluorescence microscopy are consistent with the data previously reported showing a transient cytoplasmic localization of Gcn5 (Dastidar et al., 2012) as well as its presence in sub-cellular mitochondrial fraction (http://cyclops.ccbr.utoronto.ca/).

### The Gcn5 protein is present inside mitoplasts

The result of the fluorescence experiment prompted us to investigate the subcellular localization of the Gcn5 protein by western blot. For this purpose, we used the W303-1A strain genomically expressing the protein with the C-terminal myc-epitope (Vernarecci et al., 2008). Cells expressing, or not expressing, GCN5-9Myc grown in glycerol or in glucose containing media were fractionated by differential centrifugation in mitochondrial and cytosolic fractions. Western blot analysis was performed by hybridization with anti-myc antibody; anti-Ada2 (nuclear) and anti-Por1 (mitochondria) antibodies were used to control the quality of fractions.

**Fig. 6.**
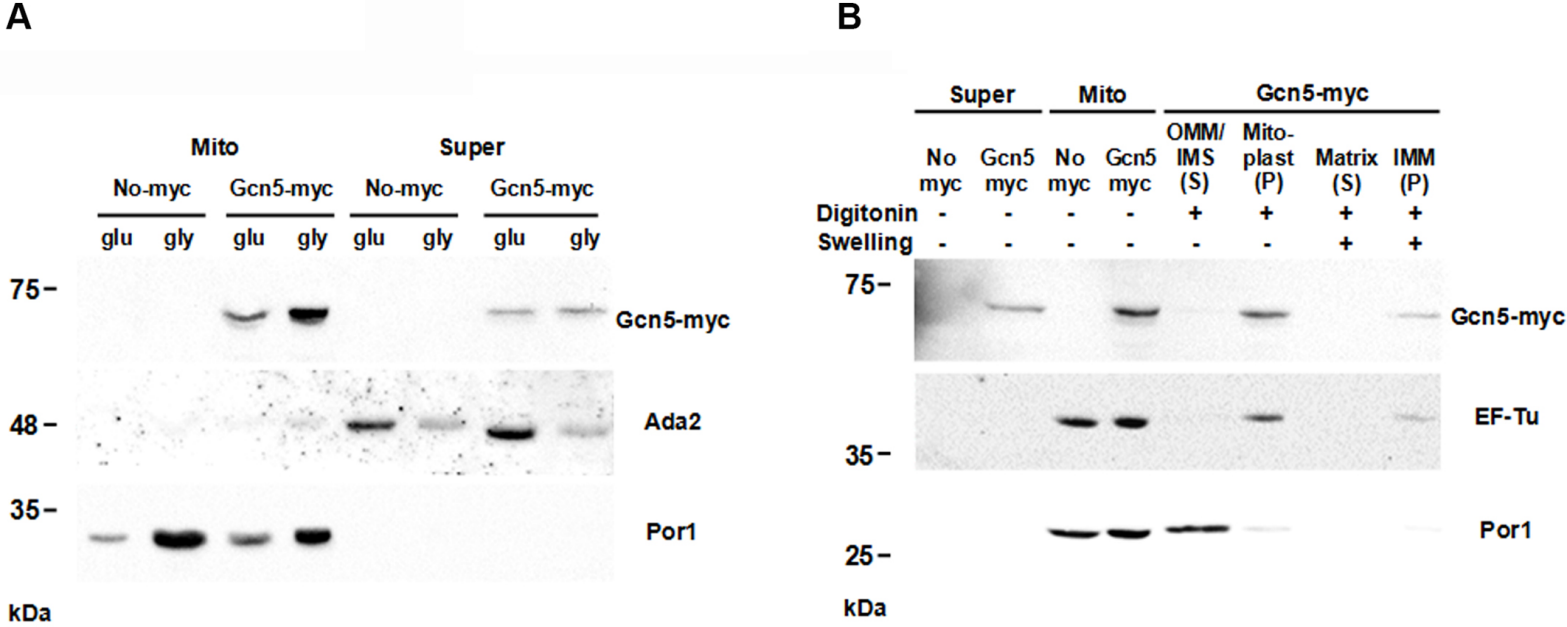
**Subcellular and submitochondrial fractions indicate that Gcn5 protein is localized in the mitoplasts.** (A) Whole cell lysate (Super) and mitochondrial (Mito) fractions of W303-1A GCN5-9Myc or untagged control (No-myc) strains grown in YP 2% glucose or 3% glycerol containing media were analysed by 10% SDS PAGE. Gcn5-myc, Ada2 and Por1 antibodies were used as nuclear and mitochondrial marks, respectively. (B) Purified mitochondria (Mito) and supernatant (Super) from rho^+^ cells were treated (+) or not (−) with Digitonin or swelling buffer and sonication (Swelling) to obtain mitochondrial fractions. After isolation of outer membrane and inner membrane space (OMM/IMS), mitoplasts were fractionated in inner membrane (IMM) and matrix. Pellet (P) and soluble (S) fractions were isolated by ultra centrifugation and immunostained with antibodies against the Gcn5-myc, EF-Tu and Por1 for marks of different mitochondrial compartments (see Materials and Methods for details).

Fig. 6A shows a consistent presence of Gcn5 protein in the mitochondrial fraction. This was not the case for another SAGA subunit (Ada2); this result suggests a specific mitochondrial localization of Gcn5 independently from SAGA complex. We have previously shown that Gcn5 protein is overexpressed in respiring cells compared to cells grown in glucose containing medium (Canzonetta et al., 2016). Here, we show that this increase also involves the mitochondrial fraction of the protein (Fig. 6A).

To assess the Gcn5 distribution within mitochondria, purified mitochondria from rho^+^ and rho° cells expressing, or not expressing, GCN5-9Myc were fractionated into soluble and particulate (pellet) fractions after digitonin treatment, which results in disruption of the mitochondrial outer membrane. Mitoplasts were fractionated in inner mitochondrial membranes and matrix (see Materials and Methods). The quality of separated fractions was checked by hybridizing the filter with specific antibodies for Por1 (as an integral protein of outer membrane) and for EF-Tu as a mitoplast mark (Ling et al., 1997).

The results shown in Fig. 6B indicate that in WT mitochondria, the Gcn5 protein is inside the mitoplasts and is localized in the inner mitochondrial membrane. The same mitochondrial localization of the Gcn5 protein is observed in rho° cells (Fig. S2), suggesting an interaction between Gcn5, mtDNA and membrane protein(s).

## DISCUSSION

In this paper we demonstrate that the Gcn5 protein is present in yeast mitochondria independently from the SAGA complex; moreover the protein has a direct or indirect role in mtDNA maintenance. All data concerning growth capability on glucose or glycerol containing media, microscopic localization, western blot analysis of cell fractions and genetic analysis are consistent for an important role of Gcn5 in mitochondria.

Considering the multifaceted activity of Gcn5, perturbing its cellular level is expected to affect multiple cellular networks and pathways. The presence of Gcn5 in mitochondria is investigated here for the first time and sheds new light on the functioning of this versatile protein as nuclear gene transcription regulator as well as mitochondrial factor.

Our results suggest a new role for the Gcn5 protein, distinct from the catalytic activity performed on histone acetylation, e.g., acetylation of mitochondrial factors may affect their binding to the DNA or alter the protein turnover. On the other hand, *GCN5* deletion produces a decrease in mtDNA copy number and deletion of regions of the molecule (Figs 3 and 4). This suggests that an interaction of Gcn5 protein takes place at specific sites of the mtDNA.

Figs S1 and S2 demonstrate that Gcn5 is present in mitochondria even in rho° cells in which the mtDNA is absent. Therefore, we could hypothesize that Gcn5 has a specific interaction with mtDNA, but also interacts with protein(s) probably present in the mitochondrial inner membrane.

Several nuclear gene variants may influence mitochondria efficiency. Kirchman et al. (1999) have demonstrated that retrograde regulation is constitutively active in W303-1A strain, whereas we have suggested that in D273-10B/A1 cells is absent or strongly reduced (De Luca et al., 2006). Gcn5 is also involved in mitochondrion–nucleus communication as a component of the SILK complex that contains Rtg2 (retrograde transcriptional activator) (Jazwinski, 2005).

The difference observed between the two *gcn5*Δ strains (Fig. 1) might be related to the mtDNA organization/composition. Interestingly the mitochondrial genome of D273-10B/A1 cells is smaller compared to W303-1A, due to the absence of several introns in genes coding for the mitochondrial respiratory subunits and due to differences in the number of G+C clusters. Here we show that in strain W303-1A, but not in D273-10B/A1, the deletion of *GCN5* results in the loss of mtDNA. This may show a new function of the Gcn5 protein, which might be involved in the interaction of mtDNA with mitochondrial membranes. In other words, we suggest that the different structure of the mtDNA in the studied strains may influence the Gcn5 protein activity on the nucleoid structure and/or its interaction with membranes. This hypothesis is supported by the results of DAPI staining (Fig. 3A) which shows a different number and morphology of mtDNA fluorescence dots in the two *gcn5*Δ strains.

The mtDNA is organized in nucleoids, composed of a set of DNA-binding core proteins involved in mtDNA maintenance and transcription, and a range of peripheral factors, which are components of signalling pathways controlling mitochondrial biogenesis, metabolism, apoptosis and retrograde mitochondria-to-nucleus signalling. The number of mtDNA molecules contained within a single nucleoid is still an open question; nucleoids are attached to the mitochondrial inner membrane and do not appear to freely diffuse within the matrix compartment of the mitochondrion. It is possible that Gcn5 is present in nucleoids. This statement is strongly supported by the results of Fig. 3 and raises a series of important questions related to the structure and relationships between mitochondrial membranes, mtDNA and to the structure and organization of the nucleoids. Future studies on the quantitative regulatory aspects of factors involved in the binding of mtDNA to the membranes might give some important clues to the structure of nucleoids unravelling a possible new function of Gcn5 in mitochondria.

The present results, which demonstrate the presence of the Gcn5 protein inside mitochondria, suggest two different hypotheses. Gcn5 might retain its transacetylation function and hence might be important for mitochondrial transcription. This could be very relevant because no evidence has been available until now on mitochondrial transcription and its regulation.

Alternatively, Gcn5 might have a new function and be involved in the interaction of mtDNA with mitochondrial membranes. We could hypothesize an interaction between Gcn5 and mtDNA mediated by other mitochondrial proteins probably localized on the mitochondrial membrane. This aspect has been studied in the *gcn5*Δ subclones reported in Table 2 by western blot using Por1 as a marker of mitochondrial membranes (Fig. S3). The decrease of the Por1 protein in the *gcn5*Δ strains is consistent with the loss of mtDNA here described.

The mitochondrial localization of a protein lacking the targeting sequence might be considered somewhat surprising. However about one half of the over 1 thousand mitochondrial nuclearly encoded proteins has been reported to lack a classical mitochondrial import pre-sequence and internal targeting signals certainly exist (Bolender et al., 2008); among them, the human GCN5L1 homologous to *GCN5* (Inoue et al., 1996) has been reported as a component of the mitochondrial acetyltransferase machinery (Scott et al., 2012). For Gcn5, the mitochondrial targeting signals should be further investigated together with the possible post-translational modifications of the mitochondrial Gcn5 fraction.

## MATERIALS AND METHODS

### Yeast strains and growth conditions

*S. cerevisiae* strains used are:

W303-1A (*MATa ade2-1, trp1-1, leu2-3, 112, his3-11,15, ura3 can1-100 ssd1*) and its derivatives: gcn5Δ (*MATa ade2-1, trp1-1, leu2-3,112, his3-11,15, ura3 can1-100 ssd1. gcn5::KanMX4*) rho^+^ (Thomas and Rothstein, 1989; Ornaghi et al., 2005) and rho°, and GCN5-9Myc *(MATa ade2-1, trp1-1, leu2-3112, his3-11,15, ura3 can1-100 ssd1. GCN5-9Myc-klTRP1)* rho^+^ (Vernarecci et al., 2008) and rho°. The rho° strains were obtained by Ethidium Bromide treatment (Montanari et al., 2008).

W303-1B (*MATα, ade2- 1, trp1-1, leu2-3,112, his3-11,15, ura3-1 can1-100*) rho^+^ and rho° (Thomas and Rothstein, 1989; Montanari et al., 2014).

D273-10B/A1 (*MATα, met6, ura3-52*) (Sherman and Slonimski, 1964) rho^+^, rho° and its derivative *gcn5*Δ strain were obtained by replacement with the *kanMX4* marker gene using the long flanking homology (LFH) procedure (Wach, 1996).

GCN5-GFP clone: yeast GFP-Clone YGR252W (Life Technologies) in which the nuclear GCN5 gene was fused at 3′end with the GFP gene (Huh et al., 2003).

Yeast cells were grown at 28 or 37°C in YP medium (1% yeast extract, 1% bactopeptone) containing 2% glucose or 3% glycerol and 1.5% agar for solid medium. Comparison of growth capability was investigated by serial dilutions of concentrated suspensions (5–9×10^6^ cell ml^−1^), prepared from fresh single colonies, spotted onto a unique plate. Pictures were acquired after 2–3 days of growth on glucose plates and after 5 days of growth on glycerol plates.

Crosses and tetrad analysis were performed as in Fiori et al. (2000).

### Plasmids and transformation experiments

DNA manipulation, restriction enzyme digestion, plasmid preparations as well as *Escherichia coli* and yeast transformations were performed as described by Sambrook et al. (1989).

pmtRFP is the centromeric plasmid pYX142 in which the gene coding for the Red Fluorescent Protein (fused at the 3′end with the mitochondrial targeting sequence of subunit 9 of the *Neurospora crassa* ATP-ase) is cloned (Westermann and Neupert, 2000).

### mtDNA quantification

In order to quantify the mtDNA copy number in our samples, qRT-PCR was performed. Standard curves were constructed using pJM2 vector containing the *OXI1* (Mulero and Fox, 1993) and pAXII-14 vector containing the actin gene of *Kluyveromyces lactis* (Tizzani et al., 2007). The latter gene is highly similar to *S. cerevisiae ACT1* (http://gryc.inra.fr/) and it has been used as housekeeping gene for quantification of nuclear DNA copy number in our cells. Plasmids were carefully quantified and serial dilutions starting from 1.06×10^+07^ copy number and ending at 8.48×10^+04^ copy number for *ACT1* standard curve and from 5.60×10^+06^ copy number and ending at 4.48×10^+04^ copy number for *OXI1* standard curve were used. The experiments were carried out in the Rotor-Gene Q apparatus from Qiagen. The samples were prepared in duplicate by adding to 50 ng of total DNA 12.5 μl of the reaction mixture, containing using SensiMix^TM^ SYBR No-ROX Mastermix (Bioline) and 0.3 μM of the oligonucleotides OXI1+ and OXI1− for mitochondrial gene amplification or ACT1+ and ACT1− for nuclear gene amplification (Table 1); H_2_O was added to reach the final volume of 25 μl. The ratio between ACT1 mean value and OXI1 mean value was used to overcome the variability among samples caused by DNA quality and DNA quantification errors.

**Table 1.**
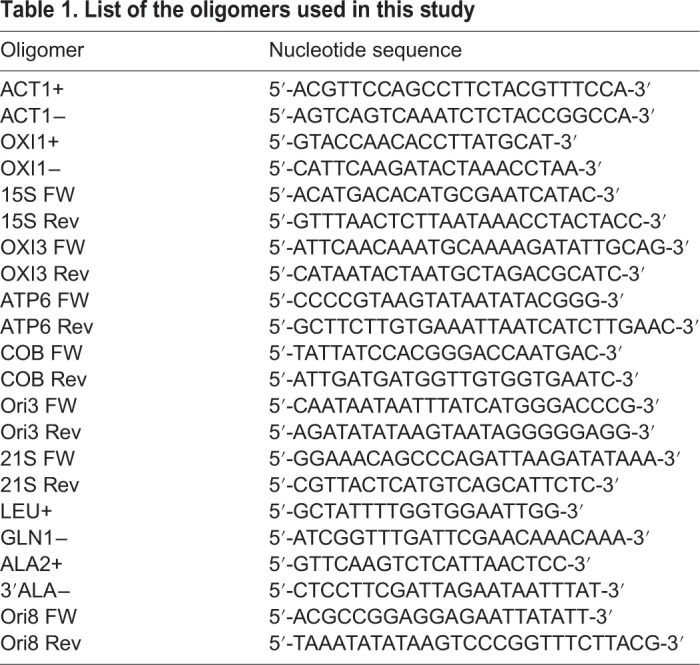
**List of the oligomers used in this study**

**Table 2.**
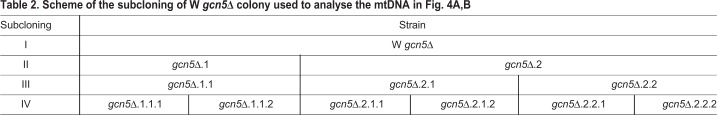
**Scheme of the subcloning of W *gcn5*Δ colony used to analyse the mtDNA in Fig. 4A,B**

To amplify the mtDNA we performed the colony PCR, treating a single fresh colony grown on YP 2% glucose plate with 100 μl of 0.2 M CH_2_COOH, 1% SDS for 15 min at 70°C and then adding 300 μl of EtOH. After vortexing the mixture was centrifugated for 3 min at 13,000 rpm and resuspended in 100 μl of TE 1X; 3 μl was used as template for PCR. The thermocycler conditions were: 5 min at 95°C for one cycle, followed by 35 cycles of 30 s at 95°C, 45 s at 59°C, 1 min at 72°C and at the end 5 min at 72°C for one cycle. 10 μl of 25 μl of PCR reaction was loaded on TBE 1X, 2.5% agarose gel for electrophoretic analysis. The nucleotide sequences of the oligomers used for the colony PCR are reported in Table 1.

### Protein preparation and western blotting

Yeast cells were grown in YP 2% glucose or 3% glycerol containing media at 28°C and collected at exponential phase. Proteins were extracted from cytoplasm and from purified mitochondria (Baldacci and Zennaro, 1982). The digitonin treatment was performed resuspending 2–3 mg ml^−1^ of mitochondria in 10% glycerol, 30 mM HEPES-KOH, pH 7.4, 50 mM KAcetate, pH 7.4 and 1% digitonin. The samples were incubated for 30 min on ice and then separated into a pellet (mitoplasts) and supernatant (outer mitochondrial membrane and intermembrane space) for 30 min at 25,000 rpm using a SW50 rotor (Beckmann). To separate the inner mitochondrial membrane (IMM) from the matrix we resuspended the mitoplasts in the Swelling buffer (HEPES 20 mM, pH 7.4, TrisHCl, pH 7.4) and bath-sonicated the mixture three times at 40 kHz for 3 min followed by 3 min on ice. The pellet (IMM) and the supernatant (matrix) were separately collected by ultracentrifugation for 45 min at 25,000 rpm. Proteins of supernatant fractions were precipitated adding TCA 5% final concentration and collected by spinning down at 13,000 rpm. Samples were resuspended in 2× Laemmli buffer, loaded on 10% SDS-PAGE and blotted on nitrocellulose membranes (Amersham). Proteins were detected with primary antibodies anti-Ada2, anti-myc, anti-EFTuM (Santa-Cruz) and anti-Por1 (Life Technologies) by Long Lasting Chemilumiscent Substrate (EuroClone) and visualized by ChemiDoc™ MP Imaging System (Biorad). Additional information about antibodies is reported in Table S1.

### Fluorescence imaging

To visualize the mtDNA, the cells were grown up to the exponential phase in YP liquid medium containing 2% glucose and then fixed with 1% formaldehyde and treated with 1 μg ml−1 of DAPI stain.

For colocalization experiments, the stationary culture of the GCN5-GFP clone (Life Technologies) transformed with pmtRFP was grown in YP 2% glucose or 3% glycerol containing media at 28°C.

Cells were visualized by fluorescence microscopy on Zeiss Axio Imager Z1 Fluorescence Microscope. Images were acquired using Axio-Vision 4.8 Digital Image Processing System and objective lens 63× oil.

All experiments performed in this paper were repeated independently at least three times.

## Supplementary Material

Supplementary information
