# Supplementary material for: Gcn5 histone acetyltransferase is present in the mitoplasts
Source: Biol Open. 2019 Feb 15;8(2):bio041244. doi: 10.1242/bio.041244 (PMC6398455; doi:10.1242/bio.041244)
Supplement: Supplementary information [file biolopen-8-041244-s1.pdf]

Supplementary Information

Materials and Methods

Table S1

Antibody	Catalogue number	Dilution	Reference
Anti-Ada2	sc-6651	1:1000	Canzonetta et al., 2016
Anti-myc	sc-40	1:1000	Canzonetta et al., 2016
Anti-EFTuM	sc-393924	1:500	Niu et al., 2017
Anti-Por1	459500	1:10000	Leo et al., 2018

Real time RT-PCR

Quantitative RT-PCR (qRT-PCR) experiment was performed as described in Canzonetta et al., (2016). After overnight growth in YP liquid medium containing 2% glucose the cells were collected. Total RNA extraction, cDNA generation, sample preparation and amplification reaction are as described in De Luca et al., (2006). In order to quantify the concentration of the target transcripts in our samples 5 ng of cDNA were analyzed in each well. Standard curves were constructed using serial dilutions of WT cDNA. The experiment was carried out on a Rotor-Gene Q apparatus from Qiagen. *GCN5* transcript was measured at least in three independent experiments against *ACT1* transcription level.

Oligonucleotides used:

GCN5 Fw: 5′- CATCTTTCCATGGCTGTCATTA -3′.

GCN5 Rev.: 5′- GAACTGATGGCACAGAAAACAA -3′.

ACT Fw: 5′- ACGTTCCAGCCTTCTACGTTTCCA -3′.

ACT Rev.: 5′- AGTCAGTCAAATCTCTACCGGCCA -3′.

Biology Open • Supplementary information

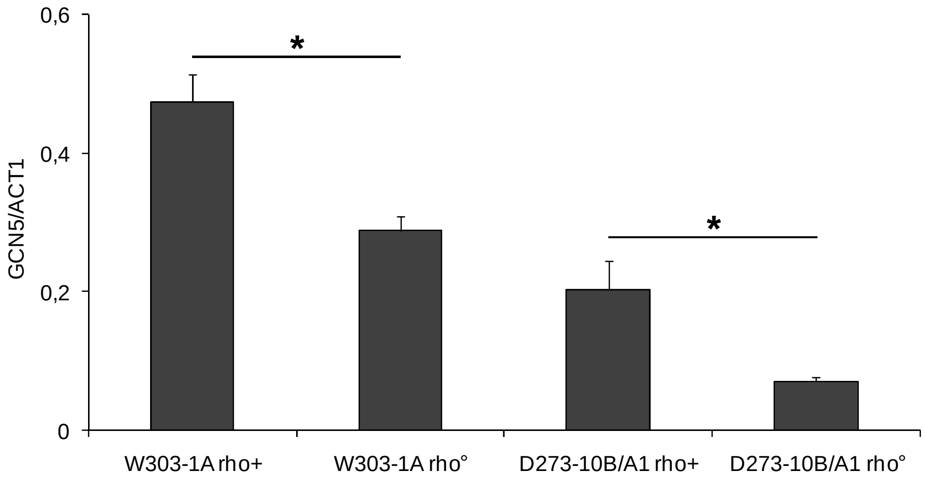

Fig. S1. *GCN5* expression is decreased in rho° cells compared to their corresponding rho$^+$. *GCN5* transcription level evaluated by qRT-PCR of the two WT W303-1A and D273-10B/A1 rho$^+$ and of their derivative rho° cells grown in YP 2% glucose containing medium. In the graph the transcription level of *GCN5* is measured as ratio of *ACT1* transcription level to overcome the variability among samples caused by RNA quality and RNA quantification errors and expressed as ng of starting quantity. The decrease of *GCN5* mRNA in rho° compared to rho$^+$ cells not only in W303-1A but also in D273-10B/A1 certainly is not responsible for the different phenotype of the two strains. Indeed, in the absence of *GCN5*, W303-1A cells have respiratory defect whereas the D273-10B have not. Means and standard error derives from at least three independent experiments and statistical significance by Student's t-test is indicated (*$p < 0.05$ for rho° versus rho$^+$ cells).

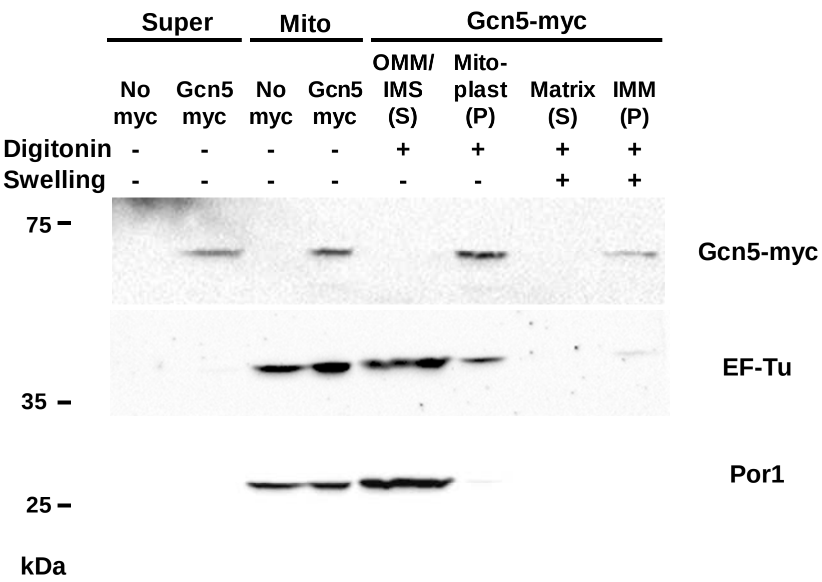

Fig. S2. Subcellular and submitochondrial fractions of rho° cells. Whole cell lysate (Super) and mitochondrial (Mito) fractions of W303-1A GCN5-9Myc rho° or untagged control (No-myc) strains grown in YP 2% glucose containing medium were analysed by 10% SDS PAGE. Mitochondrial fractions were obtained treating (+) or not (-) with Digitonin or swelling buffer and sonication (Swelling). After isolation of Outer Membrane and Inter Membrane Space (OMM/IMS), Mitoplasts were fractionated in Inner Membranes (IMM) and Matrix. Pellet (P) and soluble (S) fractions were isolated by ultra centrifugation and immunostained with antibodies against the Gcn5-myc, EF-Tu and Por1 for marks of different mitochondrial compartments (see Materials and Methods for details).

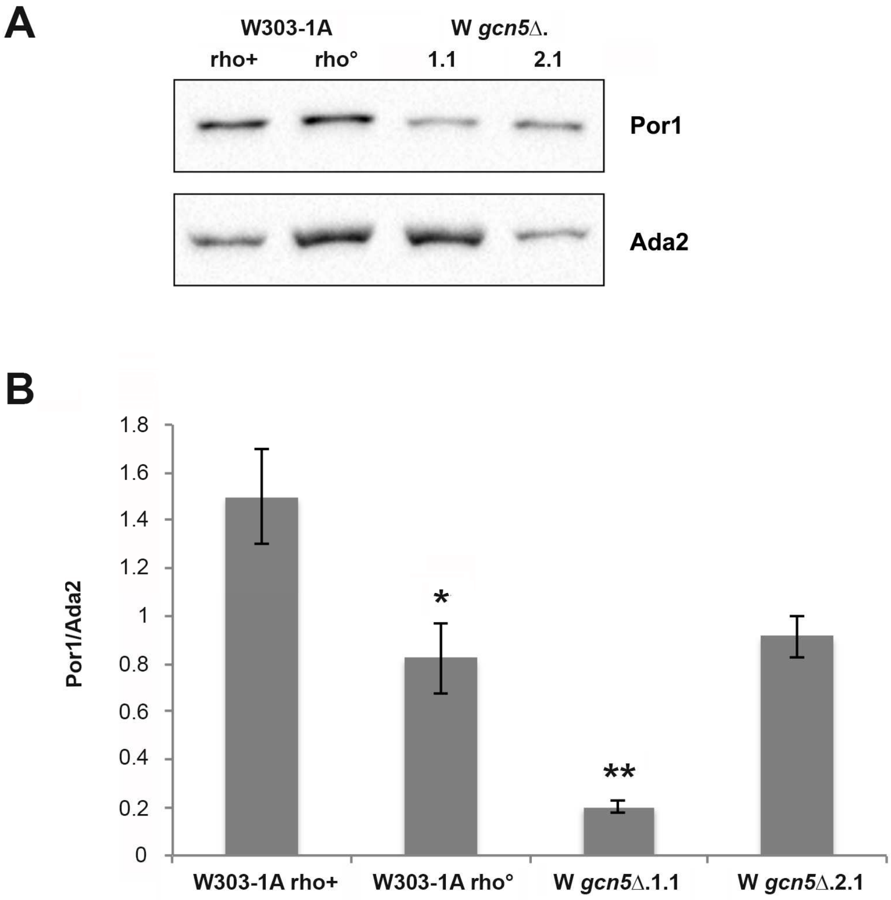

Fig. S3. Deletion of *GCN5* produces a strongly decrease of mitochondrial membranes. Panel A: Western blot analysis of total protein extracts from WT W303-1A rho$^+$, from the derivative rho° and from two W *gcn5*Δ subclones of Table 2 grown in YP 2% glucose containing medium. The protein extraction was performed as described by Canzonetta et al. (2016). Panel B: Histogram of Por1 expression normalized to Ada2 loading as control. Means and standard error derives from at least three independent experiments and statistical significance by Student's t-test is indicated (**p < 0.01; *p < 0.05).

Biology Open • Supplementary information
